# The Challenge of Fostering Healthy Organizations: An Empirical Study on the Role of Workplace Relational Civility in Acceptance of Change and Well-Being

**DOI:** 10.3389/fpsyg.2016.01748

**Published:** 2016-11-21

**Authors:** Annamaria Di Fabio, Marco Giannini, Yura Loscalzo, Letizia Palazzeschi, Ornella Bucci, Andrea Guazzini, Alessio Gori

**Affiliations:** ^1^Department of Education and Psychology (Psychology Section), University of FlorenceItaly; ^2^Department of Health Science, University of FlorenceItaly; ^3^Department of Education and Psychology (Psychology Section), Centre for the Study of Complex Dynamics, University of FlorenceFlorence, Italy

**Keywords:** positive healthy organizations, healthy business, workplace relational civility, acceptance of change, hedonic and eudaimonic well-being

## Abstract

The world of work in the twenty-first century is characterized by globalization, instability, and unavoidable change. Organizations need to develop a positive relational environment in the workplace thereby enabling workers to enhance their personal resources in order to face with on-going changes in the sphere of work for promoting their well-being. Against this background, the aim of this research was to examine the relationship between workplace relational civility and both acceptance of change and well-being (hedonic well-being as well as eudaimonic well-being) beyond the effect of personality traits. The following instruments were administered to 261 Italian workers: the Ten Item Personality Inventory (TIPI), the Acceptance of Change Scale (ACS), the Satisfaction With Life Scale (SWLS), and the Meaningful Life Measure (MLM). The results of hierarchical regression analyses revealed that workplace relational civility explained a percentage of incremental variance beyond personality traits in relation to acceptance of change, life satisfaction, and meaning in life. These results underscore the positive relationship between workplace relational civility and acceptance of change, hedonic well-being, and eudaimonic well-being, offering new research and intervention opportunities to meet the challenge of fostering healthy organizations.

## Introduction

The world of work in the twenty-first century is characterized by globalization, instability, and unavoidable change (Savickas, 2011; Guichard, 2013). In this postmodern era, work and well-being play a key role in the health of individuals, and it is important to verify here the effect of a negative working place on the health and well-being of workers (Sparks et al., 2001; Arenas et al., 2015; Giorgi et al., 2015, 2016; Mucci et al., 2016). The insecurity of the current world of work highlights the need to promote “healthy organizations” from a primary prevention point of view (Hage et al., 2007; Kenny and Hage, 2009; Di Fabio and Kenny, 2015; Di Fabio and Gori, 2016b). The concept of healthy organizations is in line with the most recent definition of health of the World Health Organization (1998) that replaced the old definition that stated that health was simply the absence of disease. The most recent definition noted that “health is a state of complete physical, mental, spiritual and social well-being and not merely the absence of disease or infirmity” (World Health Organization, 1998). Starting from this definition, the World Health Organization (2007) underlined the importance for organizations of developing a culture of health in the world of work both for the well-being of workers and for the health of organizations starting from a primary prevention point of view.

A focus on healthy people and healthy organizations is introduced (Macik-Frey et al., 2007; World Health Organization, 2007). Healthy people deal with many challenges in the world of work and in general have satisfying and productive work and personal lives. Flourishing, resilience, and the ability to adapt themselves to environment, characterize healthy people. Starting from a point of view that initially characterized health as the absence of diseases (biomedical perspective), the concept of work has been broadening to examine positive work environment factors (humanistic perspective) for employee health, well-being and performance (Macik-Frey et al., 2007; Raya and Panneerselvam, 2013). Thus, attention to healthy people as flourishing and resilient workers is paid as well as the role of a positive work environment in promoting employee health, in terms of well-being and good performance within an organizational positive psychology perspective (Di Fabio, 2014a; Snyder et al., 2014; Di Fabio and Kenny, 2015; Di Fabio and Gori, 2016b). The positive psychology perspective (Seligman and Csikszentmihalyi, 2000; Seligman, 2002) is a recent framework characterized by emphasis on resources and on positive functioning rather than on negative functioning. This change of focus highlights the importance of gainful employment and life (Di Fabio, 2014a, 2015b; Snyder et al., 2014) for oneself and for the organization too, supporting one's own family, for a safe work environment, for job satisfaction, and for engagement with and creating a sense of belonging to the organization and healthy business. The following factors are essential in an organizational positive psychology framework: satisfaction with one's work, the development of talents and skills, and positive interpersonal relationships (Snyder et al., 2014; Di Fabio, 2014a). The value of such a framework (Seligman, 2002; Seligman and Csikszentmihalyi, 2000) is that it increases the quality of working life and organizational effectiveness and concentrates on the importance of enhancing individual resources (Boyatzis et al., 2002; Di Fabio, 2006, 2015a; Boyatzis and Saatcioglu, 2008; Boyatzis, 2009; Di Fabio and Kenny, 2012, 2015, 2016a,b; Di Fabio and Maree, 2012; Di Fabio and Palazzeschi, 2012a; Di Fabio et al., 2012, 2013; Sartori et al., 2013; Di Fabio and Saklofske, 2014a,b; Boyatzis et al., 2015; Di Fabio and Bucci, 2015, 2016). The enhancement of individual resources impacts directly and indirectly on the professional life of individuals and the quality of work they produce. Furthermore, the quality of work seems to be associated with the quality of relationships in the workplace, highlighting the importance of such relationships in the overall life context (Blustein, 2011; Richardson, 2012). Positive organizational psychology thus underscores the need to develop positive and supportive relationships in the workplace (Blustein, 2011; Snyder et al., 2014; Di Fabio, 2015b).

The positive organizational psychology framework contains the idea of a healthy organization linked to the concept of “performance and health” (De Smet et al., 2007). Healthy organizations promote healthy business, relying on the importance of the health of individuals for organizational success in a framework characterized by a close connection between workers' well-being, organizational well-being and effective functioning (Di Fabio and Blustein, 2016a,b). A healthy organization can be defined as “one whose culture, climate and practices create an environment that promotes employee health and safety as well as organizational effectiveness” (Lowe, 2010; Ceschi et al., 2014). A healthy organization is conducive to healthy business, namely successful business (Grawitch and Ballard, 2016). Healthy business regards also the strong association between organizational profitability and workers' well-being (Raya and Panneerselvam, 2013; Arnoux-Nicolas et al., 2016). New ways of promoting good organizational relationships, including relationality (Blustein, 2011) and respectivity (Maree, 2012), can lead to healthy organizations (Di Fabio, 2014a, 2015b; Di Fabio and Kenny, 2016a). These relationships are fundamental aspects of people's lives with their focus on respect and care for oneself and for others (Di Fabio, 2014a, 2015b). This focus promotes flourishing relationships from a positive perspective and underscores the importance of a positive balance between “me,” “us,” “organization,” “people,” and “the world” (Snyder et al., 2014; Di Fabio, 2016a). This can be very useful in the construction of a new stage of social and human development marked by organizational and community maturity (Di Fabio, 2014a) characterized not simply by swinging between an egocentric position (centered on me) and an allocentric position (centered on others) but reaching a polycentric position (for mutually gain, for others and for myself) that asks for connectedness centered on reflexivity (Di Fabio, 2016a) and for the new paradigm of meaning (Di Fabio and Blustein, 2016a,b).

The following constructs are important in healthy organizations: prosocial organizational behavior (McNeely and Meglino, 1994), organizational citizenship behavior (Podsakoff et al., 1990), organizational support (Eisenberger et al., 1990), workplace civility (Di Fabio, 2015c), and workplace relational civility (WRC, Di Fabio and Gori, 2016b). This last construct refers to a new form of relational style in the workplace “characterized by respect and concern for oneself and others, interpersonal sensitivity, personal education, and kindness toward others. It also includes civil behaviors such as treating others with dignity and respecting social norms to facilitate peaceful and productive cohabitation” (Di Fabio and Gori, 2016b, p. 2). The workplace relational civility construct has three dimensions: Relational decency (RD) at work: decency-based relationships, characterized by respect for the self and others, assertiveness, ability to express convictions, and relational capacity; Relational culture (RCu) at work: politeness, kindness, good education, courteousness; Relational readiness (RR) at work: sensibility toward others (speed in understanding the feelings of others and showing proactive sensibility), ability to read the emotions of others, concern for others, delicacy, attention to the reactions of others, empathy, and compassion.

In particular, in comparison to the construct of emotional intelligence (EI), RR is a dimension of Workplace Relational Civility construct that refers to behavior in the workplace that is decent, prosocial, polite, careful, and that involves relational style patterns in line with one's involvement with others. RR in particular regards the understanding of the emotions of others quickly and easily, to demonstrate delicacy, empathy, compassion, and attention to their reactions. WRC thereby represents a larger construct that includes EI (Di Fabio and Gori, 2016b). Although, the academically accepted standard view of EI is as an ability model, we must acknowledge that there are other types of EI that are purported in the literature (Mayer et al., 2008; Stough et al., 2009). In the literature there are two main approaches to defining and measuring EI (Stough et al., 2009): EI as ability-based (Mayer and Salovey, 1997) and EI as trait-based self-report (Bar-On, 1997; Petrides and Furnham, 2000, 2001; Di Fabio et al., 2016).

The WRC can be assessed with a new “mirror” kind of measurement, the Workplace Relational Civility Scale (WRCS, Di Fabio and Gori, 2016b): the participants have to firstly describe their relationship with others, and then the relationship of others with them. This kind of measure allows a better evaluation of the interpersonal interactions helping to reduce bias and it recognizes the inconsistencies between how the person considers himself/herself during the interaction with others and how the person looks at others in their interaction with him/her. This kind of measurement enables individuals to reflect on his/her own actions and to examine the behavior of others, thereby making him/her more aware of the relational dynamics.

Organizations need to develop a positive relational environment in the workplace enabling workers to enhance their personal resources so that they can cope with the on-going changes in today's world of work and also improve their well-being (Di Fabio and Bernaud, 2008; Di Fabio and Palazzeschi, 2008, 2012b, 2015, 2016; Di Fabio, 2011, 2014a,b; Di Fabio et al., 2014; Snyder et al., 2014; Di Fabio and Gori, 2016a,b; Di Fabio and Kenny, 2016a,b).

In the present-day ever-changing and unstable world of work, acceptance of change is crucial (Di Fabio and Gori, 2016a). Acceptance of change (AC) is defined as “tendency to embrace rather than shy away from change.” AC thus stems from the belief that, in their work and other activities, people who are able to accept change often find that the change has a positive impact on their working lives and their resource levels” (Di Fabio and Gori, 2016a, p. 2). Acceptance of change has the following dimensions: Predisposition to change: people's ability to learn from change and to use change to improve the quality of their lives; Support for change: social support perceived from others when the person is facing challenges; Change seeking: people's predisposition not only to seek change, but also to accept and integrate life and work changes; Positive reaction to change: experience of positive emotions able to predispose people to experience change positively and to benefit from it”; and Cognitive flexibility: the mental ability to go through between different concepts also adopting flexible cognitive processing strategies (Di Fabio and Gori, 2016a, p. 3).

From a positive psychology perspective and thus also from a healthy organizations perspective, promoting the well-being of workers is vital (Adkins, 1999; Danna and Griffin, 1999). In particular, from a positive psychology perspective (Seligman and Csikszentmihalyi, 2000; Seligman, 2002), it is possible to distinguish two forms of well-being: hedonic well-being and eudaimonic well-being. Hedonic well-being (Watson et al., 1988) has a cognitive component in terms of life satisfaction (Diener et al., 1985) and an affective component characterized by the ascendancy of positive emotions over negative emotions (Watson et al., 1988). Eudaimonic well-being concerns the full functioning of the person (Ryan and Deci, 2001) and relates to life meaning and purposefulness (Waterman et al., 2010; Gori et al., 2015). In previous research, important associations between positive relationships in the workplace, the tendency to accept changes, and both hedonic and eudaimonic well-being emerged (Di Fabio, 2014a, 2016a).

People who participate positively in the workplace, are generally able to communicate with others with kindness and to attune their expressions and behaviors to those of others to promote a climate of mutual respect, show also the tendency to accept change and respond easier to the current challenges of our society of complexity (Rehfuss and Di Fabio, 2012; Di Fabio, 2016a,b,c). Furthermore people can benefit from a work climate of decency, respect, and awareness between people, enhancing relational management (Di Fabio, 2015b), psychological strenghts (Di Fabio, 2014a; Di Fabio and Gori, 2016b), hedonic and eudaimonic well-being in terms respectively of life satisfaction, and flourishing, authenticity and life meaningfulness (Di Fabio, 2014a, 2016a,b). So, given the importance of the concepts of relationality and civility, the present study is related to the idea of promoting the acceptance of change and well-being at work, through relationality (Blustein, 2006; Di Fabio and Maree, 2012, 2016; Maree, 2013; Di Fabio, 2015b; Di Fabio and Gori, 2016a,b).

### Aim and hypotheses

The principal objective of this research was to assess the relationship among workplace relational civility, acceptance of change and well-being (hedonic well-being and eudaimonic well-being) controlling for the effects of personality traits.

The following three hypotheses were formulated.

H1: The workplace relational civility assessed by the WRCS will add a percentage of incremental variance beyond the variance explained by personality traits in relation to acceptance of change.
H2: The workplace relational civility assessed by the WRCS will add a percentage of incremental variance beyond the variance explained by personality traits in relation to hedonic well-being (assessed by life satisfaction).
H3: The workplace relational civility assessed by the WRCS will add a percentage of incremental variance beyond the variance explained by personality traits in relation to eudaimonic well-being (assessed by meaning in life).

## Materials and methods

### Participants

Two hundred and sixty-one Italian workers of different public and private organizations in central Italy participated in the study. The participants' ages ranged from 34 to 58 years (*M* = 46.15, *SD* = 8.642). 175 workers (67.00%) of the participants were men with a mean age of 46.85 years, *SD* = 8.582 and 86 (33.00%) were women with a mean age of 44.73, *SD* = 8.638.

### Measures

#### Personality traits

The 10 Item Personality Inventory (TIPI, Gosling et al., 2003), in the Italian version by Di Fabio et al. (2016) was used to evaluate personality traits. The questionnaire was developed on the basis of descriptors of the Big Five instruments. Each item consists of two separated attributes with response options on a 7-point Likert scale ranging from 1 (*strongly disagree*) to 7 (*strongly agree*). The TIPI identifies five personality dimensions: Extraversion (E) (example of item: “I see myself as extraverted, enthusiastic”), Agreeableness (A) (example of item: “I see myself as sympathetic, warm”), Conscientiousness (C) (example of item: “I see myself as dependable, self-disciplined”), Neuroticism (N) (example of item: “I see myself as calm, emotionally stable”), Openness (O) (example of item: “I see myself open to new experiences, complex”). The Italian version of the TIPI indicated good values of reliability (*E* = 0.82; *A* = 0.78; *C* = 0.79; *N* = 0.71; *O* = 0.74; Di Fabio et al., 2016).

#### Workplace relational civility

The Workplace Relational Civility Scale (WRCS, Di Fabio and Gori, 2016b) was used to evaluate relational civility in the workplace. It is a self-report mirror instrument consisting of 26 items to assess relational civility at work. The WRCS has three dimensions: Relational readiness (RR) at work, Relational culture (RCu) at work, and Relational decency (RD) at work. The sum of these dimensions gives an overall score for workplace relational civility for each part of the WRCS (Part A and Part B) and a total score. Part A concerns the analysis of an individual's perception of himself or herself regarding a particular issue (example of item: “I was able to express my values and my beliefs calmly to others) while Part B concerns the analysis of an individual's perception of others regarding the same issue (example of item: “Others were able to express their values and their beliefs calmly to me”). The participants in the present study were asked to describe their general relationship with others over the past 3 months, and then to describe their perception of the general relationship of others with them over the same time period. The response format was a Likert scale with five responses (1 = *not at all*; 2 = *a little*; 3 = *somewhat*; 4 = *much*; 5 = *a great deal*). The Cronbach's alphas for the three dimensions for Part A were: Factor 1A = Relational readiness (α = 0.83); Factor 2A = Relational culture (α = 0.76); Factor 3A = Relational decency (α = 0.75). The factors for Part B were: Factor 1B = Relational readiness (α = 0.86); Factor 2B = Relational culture (α = 0.88); Factor 3B = Relational decency (α = 0.85). The Cronbach's alpha coefficients for the total score for Part A and Part B of the WRC were, respectively, α = 0.87 and α = 0.92.

#### Acceptance of change

The Acceptance of Change Scale (ACS, Di Fabio and Gori, 2016a) was used in the present study to evaluate the tendency of the participants to accept or move toward change. The ACS has 20 items with response options on a 5-point Likert-type scale (1 = *not at all*, 2 = *a little*, 3 = *somewhat*, 4 = *much*, 5 = *a great deal*). The scale enables five dimensions to be distinguished: Positive reaction to change (example of item: “I am able to give new meanings to the things that I have been doing for a long time,” Change seeking (example of item: “I am looking for changes in my life, even when things are going well,” Cognitive flexibility (example of item: “If necessary, it is not difficult for me to change my mind,” Predisposition to change (example of item: “Thinking about new plans is easy for me,” and Support for change (example of item: “I trust the people close to me when faced with change”).

The Cronbach's alpha coefficients for the five dimensions were α = 0.83 for Predisposition to change; α = 0.79 for Support for change; α = 0.80 for Change seeking; α = 0.75 for Positive reaction to change, α = 0.72 for Cognitive flexibility, and α = 0.88 for the overall scale.

#### Life satisfaction

The Satisfaction With Life Scale (SWLS, Diener et al., 1985) in the Italian version by Di Fabio and Gori (2015) was used to evaluate life satisfaction as hedonic well-being. The scale consists of five items (examples of items: “I am satisfied with my life,” “The conditions of my life are excellent”) on a 7-point Likert scale ranging from 1 = *strongly disagree* to 7 = *strongly agree*. The scale has a one-dimensional factorial structure. The Cronbach's alpha coefficient was 0.85.

#### Meaning in life

The Italian version (Di Fabio, 2014c) of the Meaningful Life Measure (MLM, Morgan and Farsides, 2009) was utilized to assess meaning in life as eudaimonic well-being. The questionnaire consists of 23 items on a 7-point Likert scale ranging from 1 = *strongly disagree* to 7 = *strongly agree*. The MLM identifies five dimensions: Exciting life (e.g., “Life to me seems always exciting”), Accomplished life (e.g., “So far, I am pleased with what I have achieved in life”), Principled life (e.g., “I have a personal value system that makes my life worthwhile”), Purposeful life (e.g., “I have a clear idea of what my future goals and aims are”), Valued life (e.g., “My life is significant”). The Cronbach's alpha coefficients were 0.85 for Exciting life; 0.87 for Accomplished life; 0.86 for Principled life, 0.85 for Purposeful life; 0.84 for Valued life. The alpha value for the total score was 0.85.

### Procedure and data analysis

The administration procedure involved some trained research assistant psychologists who administered collectively the instruments to groups of workers in meeting room in their workplace.

The order of administration of the instruments was controlled to counterbalance the effects of the order of measures. Participants completed an informed consent form and the instruments were administered in line with the Italian Law (Law Decree DL-196/2003). The study adhered to the latest version of the Declaration of Helsinki revised in Fortaleza (World Medical Association [WMA], 2013) with regard to ethical standards for research. They were followed and approved by the Department of Education and Psychology of the University of Florence (Italy).

The participants were told that they could withdraw from the study at any time and that there would be no payment for participating. Firstly descriptive statistics and Pearson's *r* correlations were carried out, and then hierarchical regressions were performed.

## Results

Means, standard deviations, and correlations between TIPI, WRCS, ACS, SWLS, and MLM are shown in Table 1.

**Table 1:** **Means, standard deviations and correlations between TIPI, WRCS, ACS, SWLS, and MLM**.

	***M***	***DS***	**1**	**2**	**3**	**4**	**5**	**6**	**7**	**8**	**9**	**10**
1. Extraversion	4.29	1.43	−									
2. Agreeableness	5.58	1.01	0.18^**^	−								
3. Conscientiousness	5.59	1.19	0.19^**^	0.39^**^	−							
4. Emotional stability	5.03	1.13	0.31^**^	0.47^**^	0.40^**^	−						
5. Openness	4.91	1.09	0.30^**^	0.26^**^	0.24^**^	0.31^**^	−					
6. WRCS Part A	52.58	5.95	0.09	0.32^**^	0.22^**^	0.19^**^	0.19^**^	−				
7. WRCS Part B	47.67	7.33	0.21^**^	0.24^**^	0.23^**^	0.31^**^	0.21^**^	0.40^**^	−			
8. ACS	73.45	9.34	0.30^**^	0.29^**^	0.22^**^	0.41^**^	0.53^**^	0.46^**^	0.43^**^	−		
9. SWLS	25.04	5.73	0.27^**^	0.32^**^	0.25^**^	0.37^**^	0.27^**^	0.32^**^	0.46^**^	0.40^**^	−	
10. MLM	123.18	20.71	0.24^**^	0.41^**^	0.48^**^	0.48^**^	0.37^**^	0.39^**^	0.50^**^	0.42^**^	0.41^**^	−

N = 261.

** p < 0.01. TIPI. Ten Item Personality Inventory; WRCS, Workplace Relational Civility Scale; ACS, Acceptance of Change Scale; SWLS, Satisfaction With Life Scale; MLM, Meaningful Life Measure.

Regarding the differences between the gender groups and the age groups in respect to the three dependent variables, below there are the results of the generalized linear model analyses conducted adjusting for the unbalancing of gender factor within the sample, introducing such a factor as dummy variable. Moreover within the same models we considered the age as covariate variable.

The first model takes into account the ACS, reporting significant effects both for gender *F*_(1, 257)_ = 6.538, *p* = 0.05, η^2^ = 0.03 and age *F*_(1, 257)_ = 11.482, *p* = 0.01, η^2^ = 0.04. For what concern the SWLS only the gender appears to play a significant effect *F*_(1, 257)_ = 6.840, *p* = 0.01, η^2^ = 0.03. The same happens for the MLM *F*_(1, 257)_ = 5.733, *p* = 0.05, η^2^ = 0.02. Despite their significance, all the previous effects appear to be characterized by a quite small magnitude in terms of effects dimensions, always ranging between 2 and 4% of the variance.

The results of three different hierarchical regression models are presented with acceptance of change, life satisfaction, and meaning in life as the criterion measures and with personality traits at the first step and workplace relational civility at the second step (Table 2).

**Table 2:** **Hierarchical regression**.

	**ACS**	**SWLS**	**MLM**
	β	β	β
**STEP 1**			
Extraversion	0.08	0.10^*^	0.01
Agreeableness	0.02	0.11^*^	0.11^*^
Conscientiousness	0.04	0.04	0.25^***^
Emotional stability	0.20^**^	0.14^*^	0.18^**^
Openness	0.38^***^	0.09	0.16^**^
**STEP 2**			
WRCS Part A	0.28^***^	0.10^*^	0.11^*^
WRCS Part B	0.12^*^	0.32^***^	0.30^***^
*R^2^ step 1*	0.36^***^	0.20^***^	0.38^***^
*ΔR^2^ step 2*	0.12^***^	0.10^***^	0.10^***^
*R^2^ total*	0.48^***^	0.30^***^	0.48^***^

The contributions of personality traits (first step) and workplace relational civility (second step) to acceptance of change (AC), life satisfaction (LS), meaning in life (ML).

N = 261.

* p < 0.05.

** p < 0.01.

*** p < 0.001. WRCS, Workplace Relational Civility Scale; ACS, Acceptance of Change Scale; SWLS, Satisfaction with Life Scale; MLM, Meaningful Life Measure.

When WRCS Part A and WRCS Part B were examined separately in Step 3 and Step 4, the models didn't account for a percentage greater of variance in relation to ACS, SWLS, and MLM.

As regard to acceptance of change, at the first step personality dimensions explained the 36% of the variance. At the second step, workplace relational civility added 12% of the incremental variance. The model overall accounted for 48% of the variance.

For what concerns the analysis explaining hedonic well-being as assessed by life satisfaction, at the first step personality traits accounted for 20% of the variance. At the second step, workplace relational civility added 10% of the incremental variance. The model overall accounted for 30% of the variance.

For what refers to the analysis explaining eudaimonic well-being as assessed by meaning in life, at the first step personality traits explained the 38% of the variance. At the second step, workplace relational civility added 10% of the incremental variance. The model overall accounted for 48% of the variance.

## Discussion

The aim of this research was to investigate the role of workplace relational civility in acceptance of change and in both hedonic and eudaimonic well-being beyond the effects of personality traits.

The first hypothesis was confirmed as workplace relational civility added a percentage of incremental variance beyond the variance explained by personality traits in relation to acceptance of change. A relational style characterized by respect and concern for oneself and others, interpersonal sensitivity, personal education, and kindness toward others (Di Fabio and Gori, 2016b) was related to acceptance of change (Di Fabio and Gori, 2016a). These results highlight the importance of developing a positive workplace relational environment so that workers can enhance their psychological strenghts (Di Fabio, 2014a; Di Fabio and Gori, 2016b) useful not only for managing their relationships but also for welcoming the ongoing changes in the workplace (Di Fabio, 2014a; Snyder et al., 2014; Di Fabio and Kenny, 2016a,b; Di Fabio and Gori, 2016a,b). People characterized by workplace relational civility in the workplace, can face adequately critical issues of change and achieve their organizational objectives coping with changes (Di Fabio and Gori, 2016b). The tendency to accept changes based on a positive relationality can help people to react better and to deal successfully with the complexity of the twenty-first century world of work (Di Fabio, 2016a).

The second hypothesis was also confirmed as workplace relational civility added a percentage of incremental variance beyond the variance explained by personality traits in relation to hedonic well-being (assessed by life satisfaction). The results of the present study showed that perceptions of civility in relationships in the workplace in terms of treating others and being treating by others with dignity and with respect in the interests of peaceful and productive cohabitation (Di Fabio and Gori, 2016a) correlated positively with greater satisfaction with one's own life (Diener et al., 1985). Thus, a relational style characterized by respect and caring for the self and the others in the relationships between people can foster relational well-being in the workplace (Di Fabio and Gori, 2016b). The construction of a positive workplace climate can affect also the overall evaluation of a person's life, contributing to hedonic well-being of individuals in their workplace.

Finally, the third hypothesis was also confirmed as workplace relational civility added a percentage of incremental variance beyond the variance explained by personality traits in relation to eudaimonic well-being (assessed by meaning in life). These results thus also supported the positive relationship between workplace relational civility (Di Fabio and Gori, 2016b) and eudaimonic well-being in terms of optimal functioning (Morgan and Farsides, 2009). Thus the perception of relational civility in an organizational environment seems to contribute to the identification of one's own meaning in work, encouraging the pursuing of meaningful goals in line with authentic aspects of the Self. The perception of good relationships in the workplace can thereby contribute to the efforts for self-realization of workers, fostering healthy individuals for healthy business and healthy organizations.

It is interesting to note that the Part A of the Workplace Relational Civility Scale regarding the evaluation of the perception of the self in relationships with others was linked closely to acceptance of change, while Part B of the WRC Scale regarding the evaluation of the perception of the relationship of others with oneself was linked closely to both hedonic and eudaimonic well-being. It seems thus that the perception of one's own workplace relational civility tends to be associated with a willingness and a readiness to face change effectively, relying on a deeper reflection on one's own possible relational actions. The perception of others' workplace relational civility tends to be associated with life satisfaction and optimal functioning, underlying the value of the behaviors of others and of the relational dynamics in the construction of own well-being. The awareness of one's own behavior and of the behaviors of other people in social relationships in the workplace, can help individuals to manage more effectively the ever-changing and unpredictable demands of their workplace, also developing new relational skills to overcome difficulties and find better solutions for themselves and the organizations.

Notwithstanding the present study showed the relationships between workplace relational civility, acceptance of change and both hedonic and eudaimonic well-being, the following limitations of the study should be noted. The research was conducted with a group of Italian workers who were not representative of the Italian population. Future research should therefore include workers from different geographical areas in Italy, from different types of organizations, and also incorporate samples from other countries. Furthermore future research could examine workplace relational civility in relation to other aspects of hedonic well-being such as positive affect (Watson et al., 1988) or other aspects of eudaimonic well-being like the subjective experience of eudaimonia (Waterman et al., 2010), the existential fulfillment (Längle et al., 2003), and the flourishing (Diener et al., 2010).

Despite the limitations of the study, the findings do add to the literature on the contribution of workplace relational civility to acceptance of change and to hedonic and eudaimonic well-being.

If the results of the present study are confirmed by future research, new interventions could be introduced to enhance workplace relational civility and thereby promote “healthy organizations,” particularly from a primary prevention perspective (Hage et al., 2007; Kenny and Hage, 2009; Di Fabio and Saklofske, 2014a; Di Fabio and Kenny, 2015). The endeavor of improving relational resources in the workplace could foster strengths of workers and organizations, important for increasing the tendency to accept the on-going changes in the world of work (Di Fabio, 2016a,b), to anticipate critical aspects and to early intervene to decrease possible risks (Hage et al., 2007; Kenny and Hage, 2009). Building a positive relational environment can also promote individual hedonic and eudaimonic well-being of workers in terms respectively of life satisfaction and meaning in life, strongly associated with organizational well-being.

Conceptualizing organizational relationality in terms of workplace relational civility in a preventive positive framework could thus strengthen healthy organizations, underlining the importance of developing positive and supportive relationships in the workplace (Blustein, 2011; Di Fabio, 2014a, 2015b; Snyder et al., 2014; Di Fabio and Gori, 2016b) for a safer and more decent relational work environment (Di Fabio and Blustein, 2016a,b).

## Author contributions

ADF conceptualized the study, choose the theoretical framework, and chose measures. OB helped in the collection of the data. ADF, MG, YL, LP and OB analyzed the data and wrote the methods and results. Then all authors wrote the paper together and read and revised the manuscript several times.

### Conflict of interest statement

The authors declare that the research was conducted in the absence of any commercial or financial relationships that could be construed as a potential conflict of interest.
